# Supplementary figures and images for: Tuberculosis patients face high treatment support costs in Colombia, 2021
Source: PLoS One. 2024 Apr 18;19(4):e0296250. doi: 10.1371/journal.pone.0296250 (PMC11025946; doi:10.1371/journal.pone.0296250)

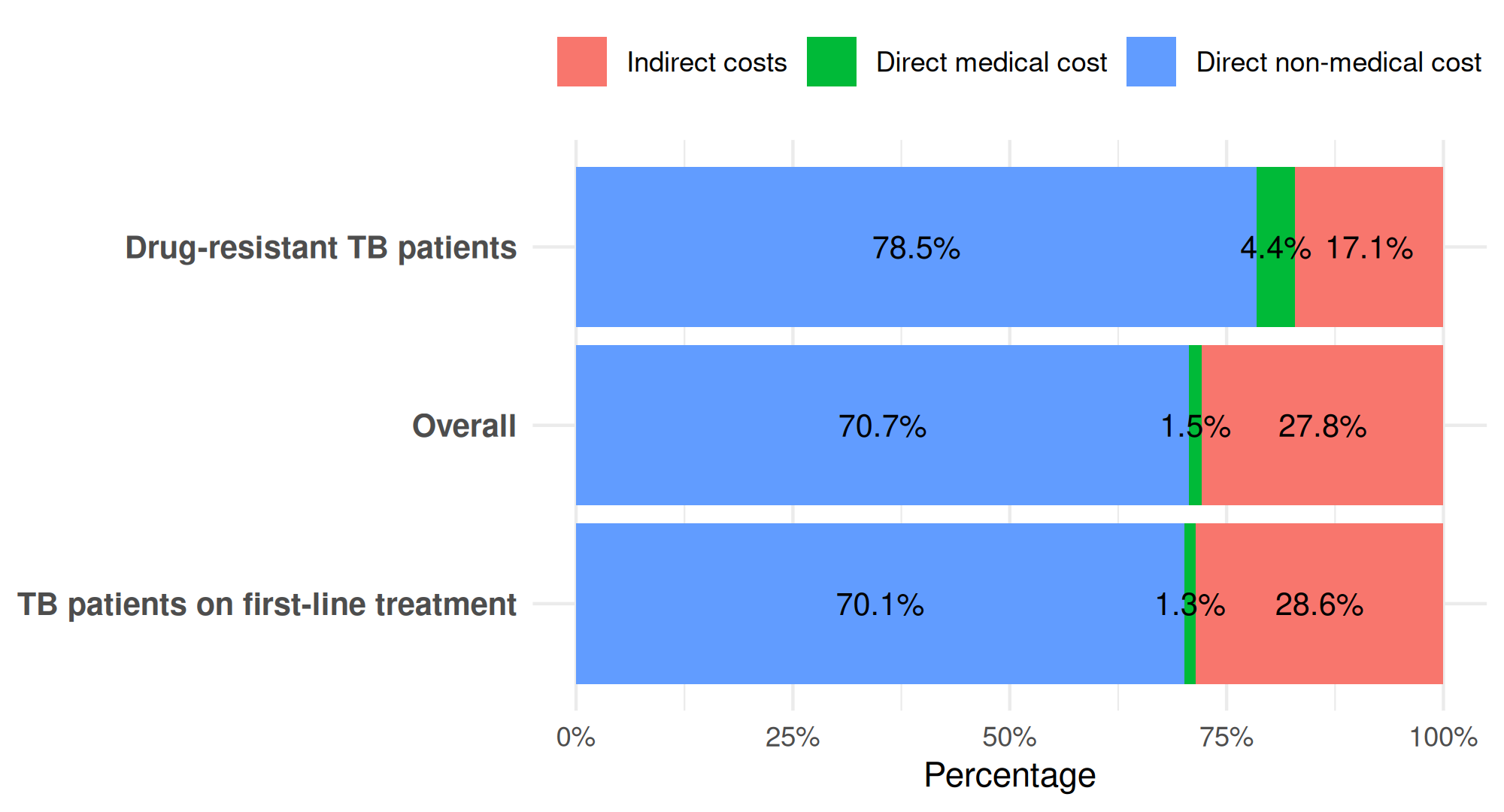

Supplement: S1 Fig — (TIF) [file pone.0296250.s002.tif]

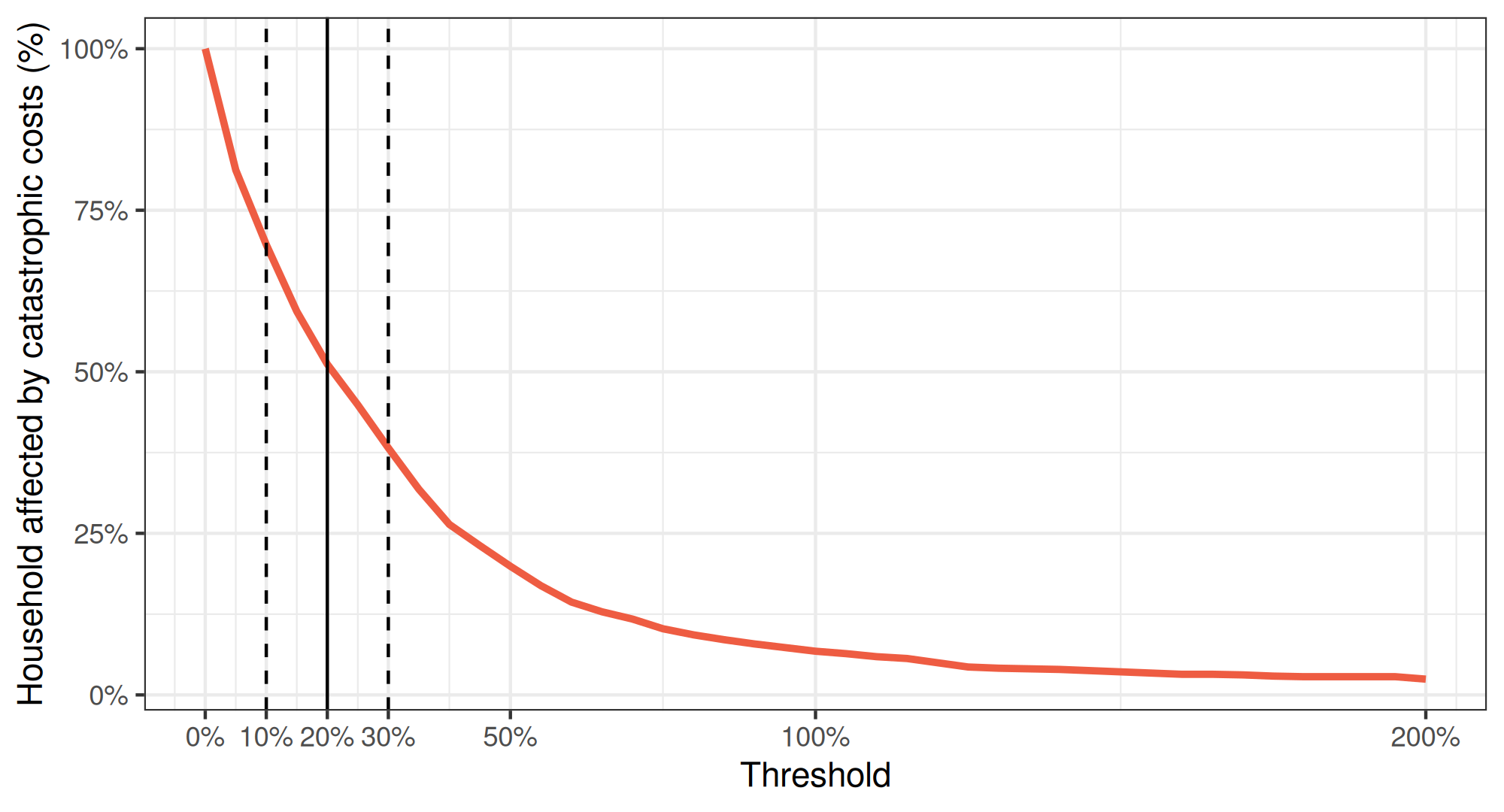

Supplement: S2 Fig — (TIF) [file pone.0296250.s003.tif]
