# Supplementary material for: Tuberculosis patients face high treatment support costs in Colombia, 2021
Source: PLoS One. 2024 Apr 18;19(4):e0296250. doi: 10.1371/journal.pone.0296250 (PMC11025946; doi:10.1371/journal.pone.0296250)
Supplement: S1 Table — *Not estimated due to low sample size. (DOCX) [file pone.0296250.s004.docx]

**Table 1S. Proportion of household that face catastrophic costs due to TB (output approach) according to different thresholds, type of costs, and quintiles of wealth, Colombia, 2021.**

| Threshold | Quintile 1 | Quintile 2 | Quintile 3 | Quintile 4 | Quintile 5 | Total |
| --- | --- | --- | --- | --- | --- | --- |
| Proportion of households experiencing direct medical, non-medical and indirect costs above various thresholds of annual income | | | | | | |
| 20% | 70.5% (63.6-77.4) | 60.9% (52.0-69.9) | 57.8% (45.8-69.8) | 41.9% (35.6-48.3) | 25.1% (19.8-30.3) | 51.7% (45.4-58.0) |
| 30% | 57.8% (49.3-66.3) | 45.7% (37.1-54.2) | 44.7% (32.8-56.6) | 26.8% (21.7-32.0) | 16% (11.1-20.9) | 38.6% (32.5-44.8) |
| 40% | 46.6% (35.3-58.0) | 31.7% (26.6-36.8) | 29.6% (21.5-37.8) | 14.1% (10.4-17.8) | 8.7% (5.2-12.3) | 26.6% (21.4-31.7) |
| 50% | 40.5% (31.5-49.4) | 23.5% (16.7-30.3) | 18% (13.6-22.4) | 10.8% (7.1-14.4) | 6% (3.2-8.7) | 20.1% (15.5-24.7) |
| 60% | 32.3% (24.2-40.4) | 17.2% (11.8-22.6) | 12.5% (8.3-16.7) | 6.6% (3.2-10.0) | 2.2% (1.2-3.3) | 14.5% (10.7-18.3) |
| Proportion of households experiencing direct medical and non-medical costs above various thresholds of annual income | | | | | |  |
| 20% | 57% (48.8-65.1) | 45.9% (39.0-52.8) | 41.7% (31.4-52.1) | 25.6% (19.8-31.4) | 14.5% (10.8-18.1) | 37.4% (31.0-43.7) |
| 30% | 44.1% (36.4-51.7) | 30.7% (25.1-36.3) | 31.8% (23.1-40.6) | 15.6% (10.1-21.2) | 8.6% (5.8-11.5) | 26.5% (21.3-31.8) |
| 40% | 35.6% (26.5-44.6) | 24.3% (19.3-29.3) | 21.9% (15.4-28.4) | 10.3% (6.1-14.4) | 6.4% (3.9-9.0) | 20% (15.6-24.5) |
| 50% | 32.1% (23.9-40.3) | 18.3% (12.2-24.3) | 16.5% (12.6-20.5) | 7.9% (4.2-11.6) | 4.3% (2.2-6.4) | 16.1% (12.2-20.0) |
| 60% | 26.9% (19.1-34.6) | 12.7% (7.1-18.3) | 11.7% (8.1-15.2) | 5.7% (2.5-8.8) | 1.4% (0.4-2.5) | 11.9% (8.5-15.3) |
| Proportion of households experiencing direct medical, non-medical and indirect costs above various thresholds of annual income (DS-TB) | | | | | | |
| 20% | 70.4% (63.5-77.2) | 61.8% (53.4-70.2) | 56.6% (43.5-69.8) | 41.5% (35.1-48) | 23.6% (18.2-29.1) | 51.3% (44.9-57.7) |
| 30% | 57.4% (49.2-65.7) | 46.3% (38-54.5) | 42.7% (29.2-56.2) | 26.3% (21.2-31.3) | 14.6% (10.3-18.8) | 37.9% (31.5-44.4) |
| 40% | 46% (34.8-57.2) | 32.1% (27.1-37.1) | 27.2% (17.8-36.6) | 13.3% (9.9-16.8) | 7.1% (4.4-9.7) | 25.6% (20.2-31.1) |
| 50% | 39.8% (31.1-48.5) | 23.8% (17.2-30.5) | 16.7% (10.4-23.0) | 10% (6.8-13.1) | 5.2% (3.1-7.2) | 19.5% (14.5-24.6) |
| 60% | 31.4% (23.5-39.4) | 17.4% (12.1-22.7) | 10.8% (4.9-16.7) | 5.8% (3.0-8.5) | 1.8% (0.8-2.8) | 13.8% (9.7-17.9) |
| Proportion of households experiencing direct medical and non-medical costs above various thresholds of annual income (DS-TB) | | | | | | |
| 20% | 57.1% (49.4-64.8) | 46.5% (39.9-53.2) | 39.6% (27.8-51.4) | 25% (19.4-30.6) | 13.1% (9.5-16.8) | 36.8% (30.3-43.3) |
| 30% | 44% (36.6-51.4) | 31.1% (25.6-36.6) | 29.1% (18.8-39.3) | 14.9% (9.9-20.0) | 7.5% (4.8-10.1) | 25.7% (20.2-31.2) |
| 40% | 35.3% (26.5-44.1) | 24.6% (19.8-29.5) | 19% (11.4-26.7) | 9.5% (5.9-13.1) | 5.2% (3.1-7.2) | 19.1% (14.5-23.8) |
| 50% | 31.7% (23.8-39.7) | 18.5% (12.6-24.4) | 15.1% (9.4-20.7) | 7.1% (4.1-10.0) | 4% (2.1-5.9) | 15.6% (11.4-19.9) |
| 60% | 26.4% (18.9-33.9) | 12.9% (7.3-18.4) | 9.9% (4.8-15.0) | 4.8% (2.3-7.4) | 1.5% (0.4-2.6) | 11.4% (7.8-15.0) |
| Proportion of households experiencing direct medical, non-medical and indirect costs above various thresholds of annual income (DR-TB) | | | | | | |
| 20% | 76.5% (34.8. 118.1) | * | 76.6% (59.8. 93.4) | 73.6% (37.5. 109.7) | 63.1% (41.9-84.2) | 65% (48.0-82.0) |
| 30% | * | * | * | * | 52.5% (37.7-67.2) | 62.2% (47.4-77.0) |
| 40% | * | * | * | * | 52.5% (37.7-67.2) | 58.8% (41.2-76.3) |
| 50% | * | * | * | * | 26.4% (0.0-65.1) | 40.5% (28.0-53.0) |
| 60% | * | * | * | * | 13.2% (0.0-32.6) | 37% (26.0-48.1) |
| Proportion of households experiencing direct medical and non-medical costs above various thresholds of annual income (DR-TB) | | | | | | |
| 20% | 50% (2.2-97.8) | * | 76.6% (59.8-93.4) | 73.6% (37.5-109.7) | 49.8% (22.9-76.8) | 57.7% (42.1-73.3) |
| 30% | * | * | * | * | 39.2% (27.6-50.9) | 54.9% (42.5-67.3) |
| 40% | * | * | * | * | 39.2% (27.6-50.9) | 51.5% (36.0-67.0) |
| 50% | * | * | * | * | 13.2% (0.0-32.6) | 33.2% (19.0-47.3) |
| 60% | * | * | * | * | * | 29.8% (16.9-42.6) |

*Not estimated due to low sample size.
