# Supplementary material for: Tuberculosis patients face high treatment support costs in Colombia, 2021
Source: PLoS One. 2024 Apr 18;19(4):e0296250. doi: 10.1371/journal.pone.0296250 (PMC11025946; doi:10.1371/journal.pone.0296250)
Supplement: S2 Table — In parenthesis 95% confidence intervals. (DOCX) [file pone.0296250.s005.docx]

**Table 2S. Total costs incurred by TB-affected households during one TB episode in Colombia, 2021, assessed by human capital method (in US$).**

| Cost category | | DS-TB | DR-TB | Total |
| --- | --- | --- | --- | --- |
| Pre-TB diagnosis | Medical | 6 (1.8-10.2) | 21.6 (0.0-57.6) | 6.4 (2.1-10.8) |
|  | Non-medical | 3.4 (0.4-6.3) | 15.6 (1.8-29.5) | 3.7 (1.0-6.4) |
| Post-TB diagnosis | Medical | 9.1 (4.3-13.9) | 101.6 (0.0-261.1) | 11.7 (6-17.4) |
|  | Travel | 284.9 (244.3-325.5) | 915.1 (64.1-1.766) | 302.5 (254.4-350.6) |
|  | Accommodation | 15.3 (0.0-33.1) | - | 14.8 (0.0-32.3) |
|  | Food | 149.7 (112-187.3) | 184.9 (65.1-304.8) | 150.6 (112.5-188.8) |
|  | Nutrition supplement | 365.4 (307.3-423.6) | 1,064 (286-1,843) | 384.9 (318.6-451.3) |
| Subtotal  Subtotal | Medical | 15.1 (9.3-20.9) | 123.2 (0.0-285) | 18.1 (11.9-24.4) |
|  | Non-medical | 822.5 (740.8-904.2) | 2,200 (1,081-3,320) | 860.9 (776.1-945.7) |
|  | Indirect costs (human capital) | 312.9 (229.8-396) | 705.6 (390.1-1,021) | 323.9 (240.3-407.5) |
| Total costs | | **1,150 (1,021-1,280)** | **3,029 (1,642-4,416)** | **1,203 (1,072-1,334)** |

In parenthesis 95% confidence intervals.
