# Supplementary material for: Tuberculosis patients face high treatment support costs in Colombia, 2021
Source: PLoS One. 2024 Apr 18;19(4):e0296250. doi: 10.1371/journal.pone.0296250 (PMC11025946; doi:10.1371/journal.pone.0296250)
Supplement: S3 Table — (DOCX) [file pone.0296250.s006.docx]

## Table 3S. Proportion of household that face catastrophic costs due to TB according to different thresholds and type of costs (human capital method).

| Threshold | DS-TB | DR-TB | Total |
| --- | --- | --- | --- |
| Proportion of households experiencing direct medical costs above various thresholds | | | |
| 20% | 0.5% (0.1-0.9) | 6.2% (0.9-11.4) | 0.6% (0.2-1.1) |
| 30% | 0.4% (0.1-0.7) | 6.2% (0.9-11.4) | 0.5% (0.1-0.9) |
| 40% | 0.2% (0-0.4) | 6.2% (0.9-11.4) | 0.4% (0-0.7) |
| 50% | 0.1% (0.0-0.3) | 3.4% (0.0-7.5) | 0.2% (0-0.4) |
| 60% | 0.1% (0.0-0.3) | - | 0.1% (0.0-0.3) |
| Proportion of households experiencing direct medical and non-medical costs above various thresholds | | | |
| 20% | 36.8% (30.3-43.3) | 57.7% (42.1-73.3) | 37.4% (31-43.7) |
| 30% | 25.7% (20.2-31.2) | 54.9% (42.5-67.3) | 26.5% (21.3-31.8) |
| 40% | 19.1% (14.5-23.8) | 51.5% (36-67) | 20% (15.6-24.5) |
| 50% | 15.6% (11.4-19.9) | 33.2% (19-47.3) | 16.1% (12.2-20) |
| 60% | 11.4% (7.8-15) | 29.8% (16.9-42.6) | 11.9% (8.5-15.3) |
| Proportion of households experiencing direct medical. non-medical and indirect costs above various thresholds | | | |
| 20% | 46.4% (40.8-52) | 70.7% (57.5-83.8) | 47.0% (41.7-52.4) |
| 30% | 33.3% (28.1-38.6) | 58.4% (48.3-68.4) | 34% (29-39.1) |
| 40% | 25% (20.7-29.4) | 58.4% (48.3-68.4) | 26% (21.9-30) |
| 50% | 20.4% (16.1-24.7) | 47.1% (34.2-59.9) | 21.1% (17.1-25.2) |
| 60% | 15.9% (12.1-19.7) | 37.5% (24.3-50.7) | 16.5% (12.8-20.2) |
