# Supplementary material for: Tuberculosis patients face high treatment support costs in Colombia, 2021
Source: PLoS One. 2024 Apr 18;19(4):e0296250. doi: 10.1371/journal.pone.0296250 (PMC11025946; doi:10.1371/journal.pone.0296250)
Supplement: Attachment — Submitted filename: Reviewer Commments.docx [file pone.0296250.s007.docx]

**Reviewer Comments**

**General comments**

1. Overall, this is a seminal and innovative work carried out by the authors exploring a very important aspect of care for TB patients primarily, with potential implication for other diseases.
2. There are no significant issues related to methodology and overall approach.
3. The authors should proof read and pay attention to language, grammar, syntax, brevity, formatting/spacing, and typos
4. The manuscript could benefit from further review by a native English speaker.

**Abstract**

1. Consider moving the definition of catastrophic cost to the methods section.
2. The last part of the last sentence of the methods section should simply read: ‘analyzed for determinants/risk factors of CC.’
3. The last sentence of the conclusion should either be specific with the categories of those at risk of CC rather the generalization: ‘vulnerable’ since there was no definition of vulnerable in the main manuscript or abstract. The conclusion section in the main manuscript is much better in this regard.

**Main manuscript**

Introduction

1. For brevity, consider removing confidence intervals in the rates reported. The reader may go to the relevant publications for the details.
2. Please clarify if the sentence: ‘Moreover, the PNPCT reports that only 71% of cases under treatment in 2020 achieved successful treatment outcomes against a target of 90%, and 10% of cases were lost to follow-up during the treatment process’ means 10% is also a target rather than an actual measure.
3. In the last sentence, consider deleting ‘cost of TB’ from the objectives as it is part of many variables in the study that aimed towards estimating CC and its determinants.

Materials and Methods

1. In the ‘Sampling’ sub-section state how individuals were selected from each of the 27 clusters. Did the authors include all the eligible subjects in the selected clusters or use some probability sampling method? Please be more explicit.

Results

1. Table 1 is too ‘busy’. Consider deleting the percentage columns and place the percentages in brackets beside the whole numbers as was done with the 95% CI in Table 2.
2. Move the comparison of rates and proportion with the study findings to the discussion section. Limit the result section to just the results.
3. For table 3, same comments applies as for Table 1 in (1) above.

Discussion

1. Limit the tendency to repeat the presentation of results in the discussion section except where it is necessary (such as comparisons).

Limitations

1. A lot of vulnerable groups were excluded based on the said ethical considerations. How this will affect the findings and conclusions therefrom should be highlighted in the limitation section.
