# Supplementary material for: Tuberculosis patients face high treatment support costs in Colombia, 2021
Source: PLoS One. 2024 Apr 18;19(4):e0296250. doi: 10.1371/journal.pone.0296250 (PMC11025946; doi:10.1371/journal.pone.0296250)
Supplement: Attachment — Submitted filename: Response letter.docx [file pone.0296250.s008.docx]

São Paulo, 28 of October of 2023

Ibrahim Jahun, MD, MSC, PhD
Academic Editor
PLOS ONE

Subject: Submission of the revised paper entitled: ***“Tuberculosis patients face high treatment support costs in Colombia, 2021”***

Dear Dr., Jahun,

We thank the reviewers for their comments on the manuscript and have edited it to address their concerns. Changes to the manuscript are shown in track changes. Our responses are given in a point-by-point manner below.

Journal Requirements:

When submitting your revision, we need you to address these additional requirements.

1. Please ensure that your manuscript meets PLOS ONE's style requirements, including those for file naming. The PLOS ONE style templates can be found at

<https://journals.plos.org/plosone/s/file?id=wjVg/PLOSOne_formatting_sample_main_body.pdf> and

<https://journals.plos.org/plosone/s/file?id=ba62/PLOSOne_formatting_sample_title_authors_affiliations.pdf>

R/. Thank you for these recommendations. We reviewed again the style requirements in order to meet them.

2. Please provide additional details regarding participant consent. In the ethics statement in the Methods and online submission information, please ensure that you have specified (1) whether consent was informed and (2) what type you obtained (for instance, written or verbal, and if verbal, how it was documented and witnessed). If your study included minors, state whether you obtained consent from parents or guardians. If the need for consent was waived by the ethics committee, please include this information.

If you are reporting a retrospective study of medical records or archived samples, please ensure that you have discussed whether all data were fully anonymized before you accessed them and/or whether the IRB or ethics committee waived the requirement for informed consent. If patients provided informed written consent to have data from their medical records used in research, please include this information

R/. Thank you for this suggestion. We included an statement in the methods section related to informed consent.

*“The approval process was backed by the Research Ethics and Methodologies Committee (CEMIN, in Spanish) of the National Health Institute of Colombia, with approval code No. CEMIN 48-2019. Verbal informed consents were recorded using the Windows Sound Recorder app and stored with an unique survey number before the interviews. Personal details that could identify participants in the study were encrypted and codified in compliance with the Habeas Data Law of Colombia. All parameters established in Resolution 8430 of 1993 were followed, thereby guaranteeing anonymity, and restricting personal details and the use and protection of personal identity (18).”*

3. Thank you for stating the following financial disclosure:

"YES. the following institution funded this study:

World Health Organization

Panamerican Health Organization

Ministerio de Salud y Protección Social

Instituto Nacional de Salud "

Please state what role the funders took in the study.  If the funders had no role, please state: "The funders had no role in study design, data collection and analysis, decision to publish, or preparation of the manuscript."

If this statement is not correct you must amend it as needed.

Please include this amended Role of Funder statement in your cover letter; we will change the online submission form on your behalf.

R/. We included the statement that you suggested in the cover letter.

*“Role of funders: The funders had no role in study design, data collection and analysis, decision to publish, or preparation of the manuscript.”*

4. Thank you for stating the following in your Competing Interests section:

"NO authors have competing interests"

Please complete your Competing Interests on the online submission form to state any Competing Interests. If you have no competing interests, please state "The authors have declared that no competing interests exist.", as detailed online in our guide for authors at <http://journals.plos.org/plosone/s/submit-now>

 This information should be included in your cover letter; we will change the online submission form on your behalf.

R/. We appreciate this recommendation. We included the following statement in the cover letter:

"No authors have competing interests."

5. We note that you have indicated that data from this study are available upon request. PLOS only allows data to be available upon request if there are legal or ethical restrictions on sharing data publicly. For more information on unacceptable data access restrictions, please see <http://journals.plos.org/plosone/s/data-availability#loc-unacceptable-data-access-restrictions>.

In your revised cover letter, please address the following prompts:

    a) If there are ethical or legal restrictions on sharing a de-identified data set, please explain them in detail (e.g., data contain potentially sensitive information, data are owned by a third-party organization, etc.) and who has imposed them (e.g., an ethics committee). Please also provide contact information for a data access committee, ethics committee, or other institutional body to which data requests may be sent.

   b) If there are no restrictions, please upload the minimal anonymized data set necessary to replicate your study findings as either Supporting Information files or to a stable, public repository and provide us with the relevant URLs, DOIs, or accession numbers. For a list of acceptable repositories, please see <http://journals.plos.org/plosone/s/data-availability#loc-recommended-repositories>.

We will update your Data Availability statement on your behalf to reflect the information you provide.

R/. We added complementary information in this sense in the cover letter:

*Publication of the study results was not contingent upon sponsor’s approval. Survey data sets contain privacy-sensitive information including participants’ individual and household income that formed a core part of the analysis. Even though we removed patient identifiers such as patient number and name, there is still a possibility that those who are familiar with the project sites and beneficiaries may be able to identify participants and their households. The informed consent recorded by all participants explicitly mentioned that only the research team have access to the data set. Due to such ethical and confidentiality restrictions, data sets will be made available only upon request and with permission from World Health Organization or by contacting the Research Ethics and Methodologies Committee (CEMIN, in Spanish; contact:* [*cein@ins.gov.co*](mailto:cein@ins.gov.co)*) of the National Health Institute of Colombia.*

6. Please review your reference list to ensure that it is complete and correct. If you have cited papers that have been retracted, please include the rationale for doing so in the manuscript text, or remove these references and replace them with relevant current references. Any changes to the reference list should be mentioned in the rebuttal letter that accompanies your revised manuscript. If you need to cite a retracted article, indicate the article’s retracted status in the References list and also include a citation and full reference for the retraction notice.


[Note: HTML markup is below. Please do not edit.]

R/. We reviewed the references of the manuscript. Thanks for this recommendation.

_________________________________________________________________________

Reviewers' comments:

Reviewer's Responses to Questions

**Comments to the Author**

1. Is the manuscript technically sound, and do the data support the conclusions?

The manuscript must describe a technically sound piece of scientific research with data that supports the conclusions. Experiments must have been conducted rigorously, with appropriate controls, replication, and sample sizes. The conclusions must be drawn appropriately based on the data presented.

Reviewer #1: Yes

Reviewer #2: Yes

2. Has the statistical analysis been performed appropriately and rigorously?

Reviewer #1: Yes

Reviewer #2: Yes

3. Have the authors made all data underlying the findings in their manuscript fully available?

The [PLOS Data policy](http://www.plosone.org/static/policies.action#sharing) requires authors to make all data underlying the findings described in their manuscript fully available without restriction, with rare exception (please refer to the Data Availability Statement in the manuscript PDF file). The data should be provided as part of the manuscript or its supporting information, or deposited to a public repository. For example, in addition to summary statistics, the data points behind means, medians and variance measures should be available. If there are restrictions on publicly sharing data—e.g. participant privacy or use of data from a third party—those must be specified.

Reviewer #1: No

Reviewer #2: Yes

4. Is the manuscript presented in an intelligible fashion and written in standard English?

PLOS ONE does not copyedit accepted manuscripts, so the language in submitted articles must be clear, correct, and unambiguous. Any typographical or grammatical errors should be corrected at revision, so please note any specific errors here.

Reviewer #1: Yes

Reviewer #2: Yes

5. Review Comments to the Author

Please use the space provided to explain your answers to the questions above. You may also include additional comments for the author, including concerns about dual publication, research ethics, or publication ethics. (Please upload your review as an attachment if it exceeds 20,000 characters)

Reviewer #1: The manuscript is well written. The findings are interesting and would go along way in supporting policymakers to address TB situation in Colombia. Please address the following observations:

1. There are several typos, please review and address them. The paper is not line numbered and I therefore cannot provide specific line references to ease your edit.

R/. Thank you for the suggestion. We reviewed the entire manuscript in order to enhance its readability. Now, the paper is line numbered.

2. It will be good to add some socioeconomic characteristics of the households because these have direct relationship with CC among the TB patients. Alternatively, consider adding table 1s in the body of the paper. This table is very important.

R/. Thank you for your recommendations. We included the reported monthly household in the table 1. Regarding to the Table 1S, this one is very busy. For this reason, it was placed on the supplementary material and described in the results section of the paper.

3. In the result section, language should be revised to ensure results are just stated and not discussed. Some sentences in the result section are more like “discussion”.

R/. Thank you very much for this useful comment. We edited the results section. Language that appears be more like “discussion” were placed in the discussion section of the manuscript.
4. It will be good to indicate how CC is distributed among 32 departments and Bogota. This level of details may be useful for policy makers to design department-based interventions thereby ensuring efficiency in resource allocation.

R/. We appreciate your suggestion. However, CC among the Colombian departments will be presented in an additional manuscript that will show some inequalities among Colombian regions. This was an express request by the Colombian Ministry of Health.

5. In table 1, HIV is indicated as clinical factors but this was not discussed. We all know that HIV is critical comorbidity among TB patients and obviously this may serve as a confounder thereby affecting CC. Additionally, the authors may need to explain how other confounders such as other chronic illnesses were addressed. Perhaps these could be attributed to limitations of the study.

R/. Thanks for this recommendation. Now, we discussed the HIV as a clinical factor in the discussion section of the paper.

Reviewer #2: Reviewer Comments
General comments
1. Overall, this is a seminal and innovative work carried out by the authors exploring a very important aspect of care for TB patients primarily, with potential implication for other diseases.

R/. Thanks for your commentary. We agree with you.

2. There are no significant issues related to methodology and overall approach.

R/. We want to thank you for your revision.

3. The authors should proof read and pay attention to language, grammar, syntax, brevity, formatting/spacing, and typos.

R/. We appreciate your suggestion. We reviewed the wording of the entire manuscript.

4. The manuscript could benefit from further review by a native English speaker.

R/. Thank you for this recommendation. This revised version was reviewed by a native English speaker.

Abstract
1. Consider moving the definition of catastrophic cost to the methods section.

R/. Thanks for this suggestion. We moved it, as follows:

***“Material and methods:*** *A nationally representative cross-sectional survey was conducted among participants on TB treatment in Colombia, using telephone interviews due to the exceptional context of the COVID-19 pandemic. The survey collected household costs (direct [medical and non-medical out-of-pocket expenses] and indirect) over an episode of TB, loss of time, coping measures, self-reported income, and asset ownership. Total costs were expressed as a proportion of annual household income and analyzed for risk factors of CC (****defined as costs above 20% annual household income****).”*

2. The last part of the last sentence of the methods section should simply read: ‘analyzed for determinants/risk factors of CC.’

R/. Thank you for this precision. We changed it as you suggested. It can be observed above.

3. The last sentence of the conclusion should either be specific with the categories of those at risk of CC rather the generalization: ‘vulnerable’ since there was no definition of vulnerable in the main manuscript or abstract. The conclusion section in the main manuscript is much better in this regard.

R/. Thanks for this commentary. We deleted “vulnerable” of this section. Now, the new conclusion section of the abstract:

*“Conclusion: Main cost drivers for CC were non-medical out-of-pocket expenses and loss of productivity (indirect costs). Current social protection programs ought to be expanded to mitigate the proportion of TB-affected households facing CC in Colombia, especially those with lower income levels.”*

Main manuscript
Introduction
1. For brevity, consider removing confidence intervals in the rates reported. The reader may go to the relevant publications for the details.

R/. Thank you for your suggestion. We removed several confidence intervals from the introduction.

2. Please clarify if the sentence: ‘Moreover, the PNPCT reports that only 71% of cases under treatment in 2020 achieved successful treatment outcomes against a target of 90%, and 10% of cases were lost to follow-up during the treatment process’ means 10% is also a target rather than an actual measure.

R/. Actually, 10% is an actual measure. We rewrote the phrase in order to avoid confusion, as follows:

*“Moreover, the PNPCT reports that only 71% of cases under treatment in 2020 achieved successful treatment outcomes (target of 90%), and 10% of cases were lost to follow-up during the treatment process”*

3. In the last sentence, consider deleting ‘cost of TB’ from the objectives as it is part of many variables in the study that aimed towards estimating CC and its determinants.

R/. Thanks for your recommendations. However, as an explicit objective of the CC-TB guideline of WHO, we consider it should be explicit in the text.

Materials and Methods
1. In the ‘Sampling’ sub-section state how individuals were selected from each of the 27 clusters. Did the authors include all the eligible subjects in the selected clusters or use some probability sampling method? Please be more explicit.

R/. Yes, the twenty-seven clusters were *randomly* sampled with probability proportional to the number of persons eligible. We clarified this issue in the methods section of the manuscript.

Results
1. Table 1 is too ‘busy’. Consider deleting the percentage columns and place the percentages in brackets beside the whole numbers as was done with the 95% CI in Table 2.

R/. Thank you for your suggestion. We placed the percentages in brackets beside the numbers for all tables.

2. Move the comparison of rates and proportion with the study findings to the discussion section. Limit the result section to just the results.

R/. We appreciate your recommendation. We moved the comparison of rates and proportions to the discussion section of the manuscript.

3. For table 3, same comments applies as for Table 1 in (1) above.

R/. Thanks. We followed previous recommendations to improve this issue.

Discussion

1. Limit the tendency to repeat the presentation of results in the discussion section except where it is necessary (such as comparisons).

R/. Thank you for this suggestion. We reviewed all the results and discussion sections in order to avoid repetitions of several results.

Limitations
1. A lot of vulnerable groups were excluded based on the said ethical considerations. How this will affect the findings and conclusions therefrom should be highlighted in the limitation section.

R/. Thank you very much for this suggestion. We agree with you. For this reason, we included it as a limitation of the study:

*“Our study has some limitations. In logistical terms, CC due to TB estimates did not consider several vulnerable groups like homeless, incarcerated, and people younger than 18 years. These groups are difficult to follow-up and legal constraints did not allow their inclusion in this study, as occurred in other Latin-American countries (35).”*

We hope that these revisions are sufficient to make our manuscript suitable for publication in PLOS One journal and look forward to hearing from you at your earliest convenience.

Cordially,

**Nelson J. Alvis-Zakzuk**

Correspondence author
